# Oxysterol-binding protein ORP6 regulates lipid metabolism and brain Aβ production

**DOI:** 10.1016/j.jlr.2025.100868

**Published:** 2025-07-25

**Authors:** Arlette A. Kasongo, Viyashini Vijithakumar, Khaled S. Abd-Elrahman, Radhika Prabhune, Lara Gharibeh, Rachel Nadeau, Isabelle Robillard, Shoshana Spring, Sabrina Robichaud, Shaza Asif, Derrick Gibbings, Kathryn J. Moore, John G. Sled, Matthieu Ruiz, Mathieu Lavallée-Adam, Stephen S.G. Ferguson, Baptiste Lacoste, Diane C. Lagace, Mireille Ouimet

**Affiliations:** 1 Department of Biochemistry, Microbiology and Immunology, University of Ottawa, Ottawa, ON, Canada; 2 University of Ottawa Heart Institute, Ottawa, ON, Canada; 3 Department of Cellular and Molecular Medicine, University of Ottawa, Ottawa, ON, Canada; 4 University of Ottawa Brain and Mind Research Institute, Ottawa, ON, Canada; 5 Department of Pharmacology and Toxicology, Faculty of Pharmacy, Alexandria University, Alexandria, Egypt; 6 Ottawa Institute of Systems Biology, University of Ottawa, Ottawa, ON; 7 Montreal Heart Institute Research Center, Montreal, QC, Canada; 8 Mouse Imaging Centre, Hospital for Sick Children, Toronto, ON, Canada; 9 Department of Medicine, Cardiovascular Research Center, New York University School of Medicine, New York, NY; 10 Department of Medical Biophysics, University of Toronto, Toronto, ON, Canada; 11 Département de Nutrition, Université de Montréal, Montreal, QC, Canada

**Keywords:** oxysterol-binding protein-like 6, astrocyte, lipid metabolism, lipid droplet, cholesterol efflux, high-density lipoprotein, amyloid beta

## Abstract

The mammalian brain is the most cholesterol-rich organ of the body, relying on in situ de novo cholesterol synthesis. Maintaining cholesterol homeostasis is crucial for normal brain function. Oxysterol-binding protein (OSBP)-related proteins (ORPs) are highly conserved cytosolic proteins that coordinate lipid homeostasis by regulating cell signaling, interorganelle membrane contact sites, and non-vesicular transport of cholesterol. Here, we show that ORP6 is highly enriched in the mammalian brain, particularly within neurons and astrocytes, with widespread expression across distinct brain regions, including the hippocampus, which is essential for learning and memory. Whole-body ablation of ORP6 (*Osbpl6*^*−/−*^) in mice resulted in dysregulation of systemic and brain lipid homeostasis, with elevated levels of brain desmosterol and amyloid-beta oligomers (AβOs). Mechanistically, ORP6 knockdown in astrocytes altered the expression of cholesterol metabolism genes, promoting the accumulation of esterified cholesterol in lipid droplets, reducing cholesterol efflux and plasma membrane cholesterol content, and increasing amyloid-beta precursor protein (APP) processing. Our findings underscore the role of ORP6 in systemic and brain lipid homeostasis, highlighting its importance in maintaining overall brain health.

Cholesterol homeostasis is essential to eukaryotic cells, with the highest concentrations in the plasma membrane and the lowest in the endoplasmic reticulum (ER). To maintain this gradient and prevent toxicity, excess cholesterol is either esterified and stored in lipid droplets (LDs) or effluxed to extracellular acceptors. This intricate balance is orchestrated by sterol-responsive transcription factors, including sterol regulatory element-binding protein 2 (SREBP2) and liver X receptor (LXRs), which regulate the expression of genes involved in cholesterol synthesis and efflux. In cholesterol-depleted states, SREBP2 drives the expression of genes responsible for cholesterol synthesis (*e.g.*, *HMGCR*) and uptake (*e.g.*, *LDLR*), while miR-33, co-transcribed with SREBP2, post-translationally represses cholesterol efflux genes (*e.g.*, *ABCA1*) (1, 2). Conversely, when cholesterol is abundant, SREBP2 is inhibited to limit cholesterol synthesis and uptake, and reduced miR-33 facilitates cholesterol efflux (1, 2). Additionally, the activation of LXR by cholesterol-derived oxysterols stimulates cholesterol efflux. Cholesterol movement from the cell membrane to the ER or from LDs to the cell surface for efflux involves both vesicular and non-vesicular trafficking mechanisms (3). Despite extensive study of these pathways, many questions remain regarding the mechanisms of cholesterol transfer between cellular compartments.

Oxysterol-binding-related proteins (ORPs) represent a family of lipid transfer proteins that facilitate non-vesicular transfer of cholesterol between lipid bilayers, thereby promoting cholesterol transport between subcellular organelles (4, 5). Conserved from yeast to mammals, distinct ORPs exhibit unique subcellular localization patterns, functions, and tissue expression profiles. ORP6, a less-characterized member of this family, is involved in a complex transcriptional cascade coordinated by SREBP2 (6). Notably, ORP6 is one of the most highly repressed targets of miR-33, and its expression is regulated by LXR activation (6). By regulating cholesterol trafficking between intracellular compartments and the plasma membrane, ORP6 plays a critical role in maintaining cholesterol homeostasis (6); however, its specific functions in lipid regulation, particularly within the brain, remain poorly understood.

The mammalian brain contains ∼25% of the body’s cholesterol, yet the blood–brain barrier restricts cholesterol exchange with circulating lipoproteins, necessitating unique mechanisms for cholesterol homeostasis (7). During central nervous system (CNS) development, both neurons and astrocytes actively synthesize cholesterol, with the highest rates occurring during postnatal myelination to support the formation of cholesterol-rich myelin (7). In adulthood, neuronal cholesterol synthesis declines, and astrocytes become the primary producers, generating two to three times more cholesterol than neurons (8, 9, 10). Beyond its structural role, cholesterol is essential for cognitive and learning processes (9), and defects in its homeostasis have been linked to aging-related cognitive decline and neurodegenerative diseases. Emerging evidence suggests that dysregulated cholesterol metabolites contribute to neurodegenerative pathologies (11, 12, 13), although the underlying mechanisms remain unclear.

Here, we show that ORP6 is highly expressed in the brains of mice and humans, localizing primarily to astrocytes and neurons. In vivo studies using ORP6-deficient (*Osbpl6*^*−/−*^) mice revealed disrupted plasma and brain lipid profiles and a marked increase in brain amyloid-beta oligomers (AβOs). At the cellular level, ORP6 knockdown in astrocytes impaired cholesterol homeostasis by reducing cholesterol efflux, increasing the accumulation of desmosterol and cytosolic LDs, and decreasing plasma membrane cholesterol. This shift promoted amyloid-beta precursor protein (APP) processing, leading to neurotoxic AβO production. Our findings identify ORP6 as a key regulator of lipid metabolism and suggest its potential as a therapeutic target for preventing lipid-driven brain Aβ production.

## Materials and methods

### Cell culture

Mouse astrocyte C8-D1A, C8-S, C8-D30 (astrocyte type I, II and III clones), neuroblastoma (N2A), and microglia (BV2) cell lines were obtained from the American Type Culture Collection (ATCC). Cells were grown and maintained in Dubelcco’s Modified Eagle Media (DMEM) (Corning, 10013CV) supplemented with 10% FBS and 1% penicillin-streptomycin (P/S) (Gibco, 15140122). All cells were maintained at 37°C and 5% CO_2_ with medium replacement every 2–3 days.

### Perfusion and tissue processing

Animals were anesthetized using isoflurane (1%–2%) in oxygen at a flow of 1.5 L/min and transcardially perfused with cold phosphate-buffered saline (PBS) at a low rate (7 ml/min) for 5 min. Animals were sacrificed at 6–8 weeks, at 16–18 weeks, and/or at 36 weeks (n = 3–8 per genotype per sex). Mouse brains were rapidly removed, and the olfactory bulb and cerebellum were deleted. Each brain was carefully hemisected: one-half was snap-frozen in liquid nitrogen for subsequent analyses and the other hemibrain was fixed in 10% formalin for 3–5 days and cryoprotected in 30% sucrose with 0.1% sodium azide (NaN_3_) until sectioning. Coronal sections of brain tissue (30 μm) were generated on a freezing Leica sliding microtome and stored in PBS with 0.01% NaN_3_ at 4°C for histological examination. Heart, liver, spleen, WAT, and kidney were collected, snap-frozen in liquid nitrogen and stored at −80°C.

### Generation and clinical phenotyping of Osbpl6^−/−^ knockout mice

All procedures at The Centre for Phenogenomics and the University of Ottawa adhered to the Animals for Research Act of Ontario and the Canadian Council on Animal Care guidelines, under protocols reviewed and approved by each institution’s Animal Care Committees. Animals were housed in individually ventilated cages with appropriate bedding, diet, and enrichment, at 21–22°C and 30%–55% humidity.

The C57BL6/N-*Osbpl6*^*em1(IMPC)Tcp*^/Cmmr mouse line was created by the International Mouse Phenotyping Consortium project (14) at The Centre for Phenogenomics using published protocols (15) and obtained from the Canadian Mouse Mutant Repository. Briefly, *Osbpl6*^*em1(IMPC)Tcp*^ (MGI:7256900) was made by electroporating C57BL/6NCrl zygotes with 8 μM of Cas9 protein (IDT) complexed with a synthetic single guide RNA (Synthego) targeting intron 9 upstream of *Osbpl6* exon 10 (ENSMUSE00001282206; gRNA_E10U) and an oligonucleotide repair template for loxP site insertion (Supplemental Table 4). Zygotes were transferred to pseudopregnant CD-1 recipients. Pups were genotyped by end-point PCR (Supplemental Table 4), and a founder with exon 10 deleted but no loxP sites was selected for backcrossing to C57BL/6NCrl mice. N1 pups were identified by PCR for the deletion allele (*Osbpl6*_wtF_F1 + *Osbpl6*_wt_R1 = 698 bp), and line establishment proceeded with heterozygous *Osbpl6*^*+/−*^ mice intercrossed to produce homozygous *Osbpl6*^*−/−*^ cohorts, with genotyping performed at 13–17 days of age. Comprehensive phenotyping followed International IMPReSS standards, with data accessible on the International Mouse Phenotyping Consortium portal (16). Grip strength tests (9 weeks) and PPI startle tests (10 weeks) followed protocol.

### Lipidomics analyses

Lipidomics analyses were performed at the Montreal Heart Institute Metabolomic platform using a validated workflow (18, 19). Frozen plasma (100 μl) or snap frozen brains samples (40 mg of lyophilized total brain) from 16-week-old WT or *Osbpl6*^*−/−*^ mice on a chow diet (n = 3 per sex and genotype) were processed in a single batch. Extracted samples (1 μl of plasma and 0.4 μl of brain) were injected into a 1290 Infinity HPLC coupled to a 6545 accurate mass QTOF MS system (Agilent, Technologies Inc.) with dual electrospray ionization (ESI) in both positive and negative modes. A Zorbax Eclipse plus column (C18, 2.1 x 100 mm, 1.8 μm) was used for lipid elution with a solvent gradient over 83 min at 40°C. MS data were processed with the MassHunter Qualitative Analysis software and an in-house bioinformatic pipeline (18), yielding 2,296 features for plasma and 2,960 features for brain samples. Lipid annotation was performed using a database of more than 500 previously identified lipids, and lipid identification from MS/MS analysis involved manual interpretation of spectra (20).

### WT versus APP brain samples

Hippocampi from 11-month-old WT and APPswe/PSEN1ΔE9 (APPswe) female mice were obtained from the Ferguson Lab at the University of Ottawa. Protein samples were lysed and homogenized in NP-40 cell lysis buffer (FNN0021, Thermo Fisher Scientific) with a protease inhibitor cocktail (539,134, Millipore Sigma), incubated on ice for 1 h, and centrifuged twice at 20,000 xg for 10 min. Supernatants were collected for analysis. For RT-qPCR, cortices were lysed in Trizol to isolate RNA. For microscopy, sections were deparaffinized, underwent antigen retrieval in citric acid (pH 6.0) at 95°C, and were blocked with 10% normal donkey serum (EMD Millipore, S30-100 Ml). Sections were incubated overnight at 4°C with anti-ORP6 (1:100, Novus NBP1-31456) and anti-ALDH1l1 (1:200, Thermo Fisher, 600-101-HBS). After washing, Alexa Fluor antibody conjugates (1:500, Invitrogen) were applied for 30 min. Imaging was performed on Aperio Versa 8 (Leica) at 20X.

### Methyl-beta-cyclodextrin (mβ-CD)-cholesterol complexes

mß-CD cholesterol was prepared as previously described (21) by adding methyl-beta cyclodextrin (Sigma, C4555-10G) and cholesterol (Sigma, C8667-5G) with a mß-CD/cholesterol molar ratio of 8:1 in plain DMEM. An Ultrasonic Bath 2.8 L (Fisher Scientific) was used to sonicate this mixture for 3 min at 37°C and was incubated on a rotator overnight at 37°C, and complexes were filter-sterilized prior to use.

### Western blot

Cells treated with control or ORP6 siRNA were washed with PBS and lysed in RIPA buffer (Tris-EDTA-EGTA, Complete protease inhibitor; Roche). Tissue samples were homogenized in RIPA buffer (ThermoFisher, 89,900), rotated at 4°C for 1 h, and centrifuged at 20,000 rpm for 20 min. Supernatants were collected and stored at −80°C. Protein samples (40–60 μg) were denatured at 95°C for 10 min, separated on 7.5% TGX Stain-Free™ FastCast™ gels (Bio-Rad, 1,610,181), and transferred to 0.22 μm PVDF membranes using the Trans-Blot Turbo System. Membranes were blocked in 5% milk in TBST for 1 h, then incubated overnight at 4°C with the following primary antibodies: anti-ORP6 (1:500, Novus Biologicals, NBP1-31456); anti-adipophilin (1:1000, Fitzgerald, 20R-AP002); anti-ACAT1 (1:200, Santa Cruz, sc-20951); anti-APP (1:1000, ThermoFisher, PA5-17829); anti-DHCR24 (1:1000, Cell Signaling, 2033S); anti-GAPDH (1:10,000, ThermoFisher, AM4300); and anti-GFP (1:1000, Abcam, ab290). Proteins were detected with HRP-based chemiluminescence using Clarity Max (Bio-Rad) or SuperSignal West Atto (ThermoFisher) on a ChemiDoc XRS + system. For extracellular protein quantification, supernatants from treated cells were centrifuged at 900 x g for 5 min, and protein was precipitated using a MeOH-CHCl_3_-H_2_O (4:1:3, v/v) mixture, then resuspended in RIPA buffer for western blotting.

### siRNA transfection

For ORP6 gene silencing, C8-D1A cells were grown for 24h to 70% confluence and transfected with 25 nM of *Osbpl6* SMARTpool On-TARGETplus siRNA (Dharmacon) using Lipofectamine RNAiMax Transfection Reagent (Invitrogen). Control samples were treated with an equal concentration of a non-targeting control sequence (ctrl siRNA) to control for non-specific effects in siRNA experiments. 24h post-transfection, an equal volume of 20% FBS culture media was added to the transfection mixture, and RNA or protein was cultured 48h post-transfection.

### PCR array gene expression profiling

Total RNA was extracted from C8-D1A cells treated with control siRNA. Reverse transcription was carried out on 500 ng RNA using the RT2 First Strand kit (Qiagen, 330,401). Quantitative real-time PCR (qRT-PCR) analyzed 84 genes related to lipoprotein signaling and cholesterol metabolism using the Mouse Lipoprotein Signaling & Cholesterol Metabolism RT2 Profiler PCR Array (Qiagen, PAMM-080ZD) according to the manufacturer’s protocol. Data analysis used ΔΔCt fold-change calculations via Qiagen's web-based software, with results deposited in the GEO Database (GSE79055).

### RT-PCR analysis

Total RNA was extracted from cells or tissue using Trizol and the Direct-Zol RNA MiniPrep Kit (Zymogen, R2052). 350 ng RNA was reverse transcribed to cDNA with the iScript Reverse Transcription Supermix (Bio-Rad, 1,708,841). Real-time PCR was performed using SsoAdvanced SYBR Green Supermix (Bio-Rad) on BioRad Connect. Expression was normalized to SRP14 and GAPDH and expressed as fold change relative to control.

### Cholesterol efflux

For ^3^H-cholesterol efflux quantification, C8-D1A cells were transfected in 24-well plates with control or ORP6 siRNA using RNAiMAX. Cells (1 x 10^5^/well) were seeded in OPTI-MEM with siRNA complexes. After 24 h, an equal volume of 20% FBS media containing 1 μCi/ml ^3^H-cholesterol (PerkinElmer, NET139001 MC) and 100 μg/ml mβ-CD-cholesterol was added. Following a 24h lipid load, media was replaced with 2 mg/ml BSA-DMEM for a 2h equilibration, then replaced with 50 μg/ml of ApoA1, HDL, or ApoE in BSA-DMEM for 4h. Supernatants were collected, and cells lysed in 0.5 M NaOH. Samples (50 μl) of both were dried on Lumaplates (PerkinElmer, 6006633), and radioactivity was measured using the Hydex Sense Plate reader (Gamble). Cholesterol efflux was calculated as % ^3^H-cholesterol in supernatant/(supernatant + cell ^3^H-cholesterol) x 100%. Fold-change efflux was calculated for each siRNA treatment relative to control (scrambled siRNA). Endotoxin-free apoA-I was synthesized in house (22), HDL was isolated by ultracentrifugation (23), and apoE was purchased from Abcam (ab50242).

### Lipid quantification by TLC

For quantification of ^3^H-cholesterol ester in BSA efflux samples, samples were neutralized with 2.5 M HCl, and lipids were extracted using the Bligh and Dyer method (24). For de *novo* cholesterol synthesis and Bloch pathway intermediates, C8-D1A cells (5 x 10^5^/well) were transfected with control or ORP6 siRNA, then labeled with 20 μCi/ml ^3^H-mevalonate (PerkinElmer NET602) for 24h. Cells were cooled at 4°C, washed with ice-cold PBS, and incubated with 10 mM of mβ-CD at 4°C for 15 min for membrane cholesterol extraction. Lipids were extracted, separated by TLC on silica gel plates with hexane/diethyl ether/acetic acid (70:30:1, v/v) for cholesterol esters or chloroform/acetone (99:1, v/v) for desmosterol. For desmosterol, plates were run 3 times and dried after each. Bands were visualized with iodine, scrapped, mixed with scintillation cocktail (EcoLite(+), MP Biomedicals), and counted for radioactivity using Tri-Carb 4810 TR (PerkinElmer).

### Fluorescence microscopy

Cells on 8-well chamber slides (Ibidi) were washed and fixed in 4% PFA (Alfa Aesar, 433689M) after treatment with 100 μg/ml mβ-CD-cholesterol. After washing, cells were blocked/permeabilized in 5% BSA/0.01% Saponin. For immunofluorescence, cells were incubated overnight with anti-ORP6 (1:500, Novus NBP1-31456) in buffer with 1% BSA/0.025% Saponin. Alexa Fluor or Alexa Fluor Plus (1:500-1:1000, Invitrogen) was applied for 1 h. Lipids were stained with BODIPY 493/503 (10 μg/ml, Invitrogen, D3922) or Filipin (1:20, Sigma, F9765-25 MG), and nuclei with DAPI (Invitrogen, D1306) or DRAQ5 (1:1000, Biolegend 424,101). Slides were mounted with DAKO media (Agilent, S302380-2) and imaged with a Zeiss LSM880 confocal using a 63X objective and Airyscan.

### Immunohistochemistry

Brain sections were mounted onto slides. After PBS washes, antigen retrieval was performed in citric acid buffer (10 mM citric acid, pH 6.0) at 95°C for 2 min 30 s, and sections were blocked and permeabilized in 10% donkey serum with 0.1% Triton X-100 for 1h30 at room temperature. Primary antibodies were applied overnight at 4°C, followed by PBS washes and incubation with secondary antibodies. Nuclei were stained with DAPI, and sections were mounted and dried. Imaging was performed at 20X on a Zeiss Axio Scan.Z1, with quantification done using FIJI software.

### Proteomics

Whole brain protein lysates from 16-week old WT or *Osbpl6*^*−/−*^ mice fed a chow diet (n = 5 WT females, n = 5 *Osbpl6*^*−/−*^ females, n = 5 WT males, n = 5 *Osbpl6*^*−/−*^ males) were subjected to tryptic digestion and mass spectrometry at the uOttawa Proteomics Resource Center, as previously described (25). Peptide and protein identifications were obtained using software from the Trans-Proteomic Pipeline (version 6.0.0) (17). Thermo RAW files were converted using msconvert (version 3) (26). The database search was performed using Comet (version 2021.01 rev. 0) (27) against the Swiss-Prot *Mus musculus* protein sequences obtained from UniProt (28) (downloaded on 2022-03-29) and their reverse sequences for False Discovery Rate (FDR) assessment. The database search was performed with a precursor ion mass tolerance of 20 ppm, considering fully tryptic peptides with a maximum of 2 miscleavages. Carbamidomethylation of cysteine was considered as a fixed modification and methionine oxidation as a variable modification. Peptide and protein identification confidence was assessed on each sample using PeptideProphet (29), ProteinProphet (30), and iProphet (31). Peptides and proteins were deemed confidently identified at an FDR < 1%.

### Protein differential expression

Protein spectral counts were normalized against the sum of spectral counts in each sample. Student’s *t-*tests were performed on normalized protein spectral counts to compare protein quantification in WT and *Osbpl6*^*−/−*^ mice. A *t* test was performed if 3 out of 5 samples had confidently identified the protein in sex-specific analyses. Resulting *P*-values were adjusted for multiple hypothesis testing with the Benjamini-Hochberg procedure.

### PIGNON analysis

PIGNON (32) was used to identify differentially expressed Gene Ontology (GO) terms when comparing protein quantification in WT versus *Osbpl6*^*−/−*^ mice and using information contained in a *Mus musculus* protein–protein interaction (PPI) network. PIGNON determined such altered terms using normalized spectral counts from the proteomics analysis and *Mus musculus* PPIs from the BioGRID database (32) (release version 4.4.208) and inferred GO terms from zenodo [DOI: 10.5281/ZENODO.21711]. GO terms annotating between 3 and 1000 proteins were considered by the PIGNON analysis. The weighted Monte Carlo sampling method for statistical assessment was used with a sampling frequency of 100,000. Differentially expressed GO terms were determined at an FDR ≤ 1%. As a negative control, a PIGNON analysis was performed on the PPI network without any quantitative data. GO terms that were uniquely identified in the analysis with quantitative data were considered significantly altered. The list of enriched GO terms and their PIGNON-derived *P*-values were submitted to Revigo (33) to summarize the significant terms and provide visualization.

### Brain magnetic resonance imaging

In this study, 16-week old WT or *Osbpl6*^*−/−*^ mice (n = 10 per sex and genotype) underwent brain imaging following transcardial perfusion with PBS, gadoteridol, and heparin, and 4% PFA, as previously described (34). After skull structures were dissected, they were post-fixed in 4% PFA and gadoteridol, and then stored in PBS with gadoteridol and sodium trinitride. Imaging was performed on a 7-T magnet using an 8-coil solenoid array, producing high-resolution 3D T2-weighted images with 40 μm voxel isotropic resolution over 13.2 h (35, 36). MRI data were analyzed for volumetric differences by registering brain images, generating consensus averages, and quantifying volume changes across 182 segmented structures (37, 38, 39). The resulting consensus average and Jacobian determinants were used to quantify volumetric differences between each MRI image and the average. The MAGeT pipeline (40) was used to segment images using a published classified MRI mouse brain atlas (41, 42) allowing for volume changes of 182 segmented structures to be calculated. ANOVA was conducted on the log Jacobian determinants to assess genotype and sex effects, with FDR corrections applied for multiple comparisons (43).

### Quantification of AβOs

The concentration of AβOs was determined using an Aggregated β-Amyloid Human ELISA kit (KHB3491, Thermo Scientific) according to manufacturer’s instructions, as previously (44, 45, 46, 47). Briefly, 50 mg of lyophilized or frozen brain protein samples were lysed and centrifuged at 4°C at 100,000g for 1h. The supernatant was collected and diluted with kit buffer before proceeding with the ELISA as per the manufacturer’s protocol. Protein was quantified using the Bradford protein assay. Finally, Aβ concentration was determined upon normalization to total protein concentration.

### Statistical analyses

Experiments were run in triplicates or more in some instances and presented as mean ± s.e.m. Statistical analyses were conducted using PRISM Version 9.1.2 and 10.0.0 software (GraphPad Software Inc), and statistical significance between groups was determined using 2-tailed unpaired Student *t* test, or 1-way ANOVA or 2-way ANOVA using multiple comparison correction as appropriate. A *P*-value of <0.05 was considered as statistically significant. For lipidomic analyses, linear regression without correction for multiple testing was performed. A threshold of *P* < 0.05 and fold-change (FC) > 1.25 or <0.8 was chosen to select the most discriminant entities between the 2 groups (WT compared to *Osbpl6*^*−/−*^) for further validation and identification using MS/MS analysis.

## Results

### ORP6 ablation alters plasma and brain lipids and brain AβOs

**Fig. 1:**
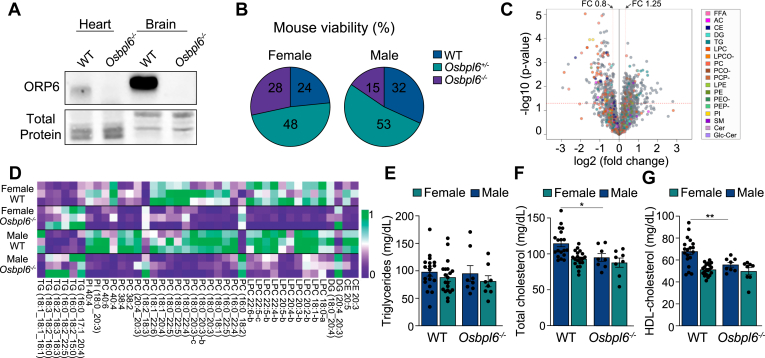
Loss of ORP6 in mice alters plasma lipid and lipoprotein profiles. A: Immunoblot of ORP6 and total protein in organs from WT and *Osbpl6*^*−/−*^ mice. B: Viability of WT, *Osbpl6*^*+/−*^ and *Osbpl6*^*−/−*^ mice shown as a percentage from heterozygous crosses. C: Volcano plot of plasma lipid altered in *Osbpl6*^*−/−*^ mice versus WT mice, with log2 fold change (FC) against -log10 *P*-value. Significance was determined by *t* test (*P* < 0.05), with FC thresholds set at <0.8 and >1.5 (n = 6 per genotype). Colored points represent significantly dysregulated lipid species by class. D: Heatmap of significantly different lipid entities annotated by MS/MS normalized to the area under the curve between WT and *Osbpl6*^*−/−*^ mice (n = 3 per genotype per sex). E: Triglyceride, (F) Total cholesterol, and (G) HDL cholesterol levels in WT and *Osbpl6*^*−/−*^ mice, stratified by sex (n = 19–20 WT, n = 8 *Osbpl6*^*−/−*^ per sex). ∗*P* < 0.05, ∗∗*P* < 0.005 by one-way ANOVA with Tukey’s multiple comparisons test (E–G).

To investigate the physiological role of ORP6, we generated *Osbpl6*-deficient (*Osbpl6*^*−/−*^) mice using CRISPR/Cas9 on a C57BL6/N background and confirmed successful knockout via Western blot analysis (Fig. 1A). *Osbpl6*^*−/−*^ mice were viable and fertile, showing no gross abnormalities. From *Osbpl6*^*+/−*^ crosses, we obtained 129 males (48%) and 141 females (52%), including 33 WT, 68 *Osbpl6*^*+/−*^ and 40 *Osbpl6*^*−/−*^ females, and 41 WT, 68 *Osbpl6*^*+/−*^ and 20 *Osbpl6*^*−/−*^ males (Fig. 1B). To explore the role of ORP6 in lipid metabolism, we performed untargeted lipidomics on plasma from 16-week-old mice. We identified 470 distinct MS signal features between WT and *Osbpl6*^*−/−*^ mice, with 40 unique lipids identified through MS/MS analysis and 54 using an in-house database (Supplemental Table 1). Several lipid classes were differentially abundant, including free fatty acids (FFA), acylcarnitines (AC), cholesterol esters, diacylglycerols (DG), and triacylglycerols (TG) (Fig. 1C and Supplemental Table 1). Notably, we observed that certain TG, DG, and PC species, particularly those enriched with 18:2 acyl chains, were increased, while cholesterol esters and several subspecies of lysophosphatidylcholines (LPCs), phosphatidylcholines (PCs), and phosphatidylinositols (PIs), which are typically enriched in high density lipoprotein (HDL) particles, were downregulated (Fig. 1D). Although there were no differences in body weight or plasma total TG levels (Fig. 1E), male *Osbpl6*^*−/−*^ mice had significantly reduced plasma total cholesterol and HDL-cholesterol levels (Fig. 1F, G).

**Fig. 2:**
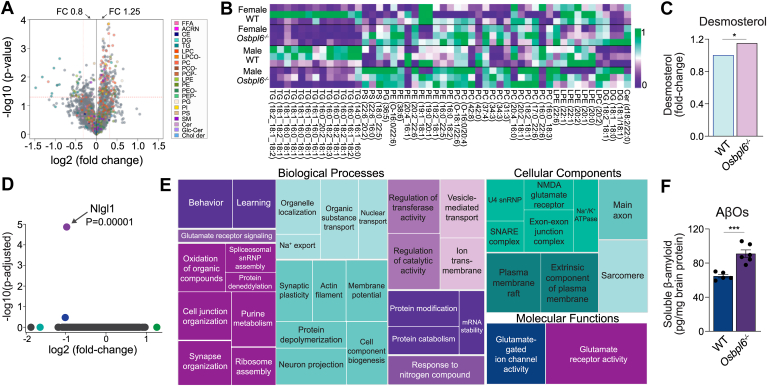
Altered CNS lipid species and biological processes in ORP6^−/−^ mice. A: Volcano plot showing altered lipid species in the brains of *Osbpl6*^*−/−*^ mice versus WT mice, with log2 fold change (FC) plotted against -log10 *P*-value. Significance was assessed using a *t* test (*P* < 0.05), with FC thresholds set at <0.8 and >1.5 (n = 6 per genotype/sex). Colored points indicate significantly dysregulated lipid species by class. B: Heatmap of significantly altered brain lipid species annotated by MS/MS normalized to the area under the curve between WT and *Osbpl6*^*−/−*^ mice (n = 3 per genotype/sex). C: Normalized MS signal intensity (Log 2) of desmosterol (mean ± s.e.m, n = 6 per genotype). ∗*P* < 0.05. D: Volcano plot of altered protein expression in male *Osbpl6*^*−/−*^ versus WT brains from unbiased proteomics analysis, showing log2 fold change against -log10 adjusted *P*-value (n = 5 per genotype/sex). E: Treemap of altered biological processes, cellular components, and molecular functions in WT and *Osbpl6*^*−/−*^ brains from unbiased proteomics analysis (n = 5 per genotype/sex). F: Whole brain amyloid-beta oligomer (AβOs) concentrations (pg/mg protein) in age-matched WT and *Osbpl6*^*−/−*^ mice at 16 weeks (n = 5 per genotype/sex, mean ± s.e.m). ∗*P* < 0.05, ∗∗*P* < 0.005, ∗∗∗*P* < 0.0005 by Student’s *t* test (C, F).

Because the blood–brain barrier is impermeable to lipoproteins, preventing lipoprotein-mediated cholesterol transport into the brain, cholesterol levels, and trafficking in the CNS are uniquely regulated by endogenous mechanisms (48). To explore the role of ORP6 in these mechanisms, we conducted unbiased lipidomics to examine the brain lipid profiles of *Osbpl6*^*−/−*^ mice as compared to WT controls. We observed that 114 brain MS signal features were distinct between WT and *Osbpl6*^*−/−*^ mice, from which we identified 51 unique lipids using MS/MS analysis, including several glycerophospholipids (GP) and glycerolipids (GP) (Fig. 2A, B and Supplemental Table 2). Notably, 3 DG species were elevated in male and female *Osbpl6*^*−/−*^ brains, while 13 TG species were reduced (Fig. 2B). Intriguingly, the cholesterol biosynthesis intermediate desmosterol was uniquely elevated in the brains of *Osbpl6*^*−/−*^ mice relative to WT (Fig. 2C). Elevated desmosterol is associated with AD, amyloid pathology and cognitive decline, making it a potential early biomarker of AD (49).

We next employed quantitative proteomics to detect proteins differentially expressed the brains of *Osbpl6*^*−/−*^ and WT mice. We identified neuroligin-1 (NLGN1), a postsynaptic cell adhesion protein critical for synaptic function (50, 51), as significantly reduced in male *Osbpl6*^*−/−*^ mice (Fig. 2D). Gene Ontology (GO) analysis revealed significant dysregulation of 42 biological processes in *Osbpl6*^*−/−*^ brains, including those related to behavior and learning, synapse organization and plasticity, and organelle localization (Fig. 2E). Go analysis of cellular components and molecular functions showed alterations in main axon and glutamate receptor activities (Fig. 2E). Notable proteins comprising these pathways included amyloid-beta precursor protein (APP), ADAM family proteases, NLGN1, and glutamate receptors (Gria1, Gria2, Gria4, Grid2) (Supplemental Table 3).

The accumulation of Aβ plaques and neurofibrillary tangles in brain regions associated with memory and cognition is a hallmark of AD (11). The oligomeric form of Aβ (AβOs) is the most neurotoxic, disrupting synaptic function, learning and memory (52, 53). Additionally, AβOs can diffuse throughout the brain, impairing neural function and contributing to AD pathophysiogy (54). Our proteomic analyses of *Osbpl6*^*−/−*^ mice revealed alterations in numerous biological processes, cellular components, and molecular functions related to Aβ production and clearance (Fig. 2E). Given the role of Aβ-regulated NLGN1 in driving synaptotoxicity in AD (51), we quantified AβOs in brain homogenates from both WT and *Osbpl6*^*−/−*^ mice using a sandwich ELISA that allows the for the specific detection of oligomeric Aβ peptides, as previously (44, 45, 46, 47). Notably, AβO levels were significantly higher in *Osbpl6*^*−/−*^ mice compared to WT (Fig. 2F). Together, these findings reveal a role for ORP6 in regulating the production of AβOs.

### Dysregulated ORP6 expression in AD and its potential role in pathogenesis

**Fig. 3:**
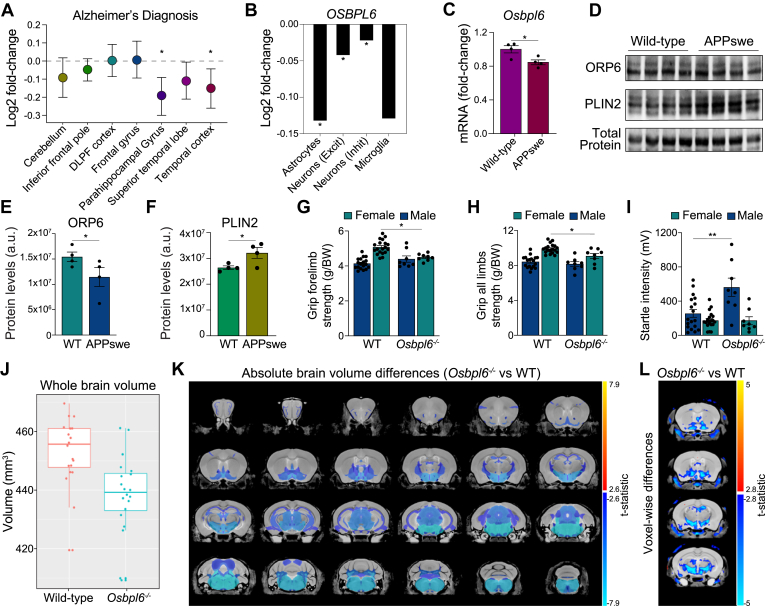
ORP6 ablation in mice leads to brain hypotrophy and impaired neuromuscular function. A: Differential expression of ORP6 between AD and control cases across brain regions, shown as Log2 fold-change ± s.e.m. using the AGORA web application. B: Differential expression of *Osbpl6* in the prefrontal cortex of AD versus healthy patients, expressed as Log2 fold change across brain cell types from snRNA-Seq data (Lau *et al.* (17)). C: qRT-PCR quantification of ORP6 mRNA in hippocampi of WT and APPswe mice (n = 4 per genotype, mean ± s.e.m). D: Immunoblotting of ORP6 and Plin2 in hippocampal tissue from WT and APPswe mice. E-F: Densitometry of ORP6 (E) and Plin2 (F) protein expression normalized to total protein (n = 4 mice/genotype, mean ± s.e.m). Grip strength of forelimbs (G) or all limbs (H) of WT and *Osbpl6*^*−/−*^ mice at 9 weeks based on the average of 3 independent trials normalized to body weight (mean ± s.e.m, n = 18–19 for WT and n = 8 for *Osbpl6*^*−/−*^). I: Prepulse inhibition startle intensity at 110 dB of WT and *Osbpl6*^*−/−*^ mice at 10 weeks (mean ± s.e.m, n = 18–19 for WT and n = 8 for *Osbpl6*^*−/−*^). J–L: Magnetic resonance imaging of WT as compared to *Osbpl6*^*−/−*^ mouse brains (n = 10 WT females, n = 10 *Osbpl6*^*−/−*^ females, n = 10 WT males, n = 10 *Osbpl6*^*−/−*^ males). Differences in total brain volume between genotypes are observed in (J), and a significant neuroanatomical effect of *Osbpl6* deletion relative to WT is visualized using *t*-statistics on a structural (K) and voxel-wise (L) level. Regions larger or smaller in *Osbpl6*^*−/−*^ mutants relative to WT are given red-yellow and blue-turquoise colours, respectively, if effects are significant at an FDR of 5%. #*P* < 0.1, ∗*P* < 0.05, ∗∗*P* < 0.005, ∗∗∗*P* < 0.0005 by Student’s *t* test (C, E, F) or by one-way ANOVA with Tukey’s multiple comparisons test (G–I).

Dysregulated cholesterol metabolism is a known risk factor for AD (10, 11, 12). In a family-based genetic study, variants of OSBPL6 (rs1347297, rs72953347) were associated with increased AD risk, although the impact on ORP6 expression remains unknown. To explore this connection, we compared ORP6 expression between healthy and AD brains. Analysis of ∼2100 publicly available human brain samples (55) revealed significant downregulation of ORP6 in the parahippocampal gyrus and temporal cortex of patients with AD relative to controls (Fig. 3A). ORP6 expression was notably lower in astrocytes, excitatory neurons, and inhibitory neurons, with the most pronounced reduction observed in astrocytes (Fig. 3B) (56). In 11-month-old transgenic APPSwe/PS1dE9 (APP/PS1) mice (45), ORP6 expression was significantly reduced at both the mRNA and protein levels in the hippocampus (Fig. 3C–E). This reduction was inversely correlated with hippocampal expression of the lipid droplet protein PLIN2 (Fig. 3D, F).

Meta-analyses suggest lower grip strength as an early indicator of AD (57, 58), a finding that has also been observed in some AD mouse models (59, 60). We observed reduced forelimb and total grip strength in female *Osbpl6*^*−/−*^ mice compared to WT controls (Fig. 3G, H). Additionally, prepulse inhibition (PPI) testing revealed an exaggerated startle response in male *Osbpl6*^*−/−*^mice (Fig. 3I), mirroring the sensorimotor gating deficits seen in AD (61). Altered neuroanatomy and reduced neuroanatomical plasticity have been reported in Aβ mouse models of AD (62). Post-mortem MRI scans of *Osbpl6*^*−/−*^ mice revealed a 3.2% reduction in brain volume (438.4 mm^3^) compared to WT controls (452.8 mm^3^) (Fig. 3J). Further analysis revealed significant structural volume reductions in 45 of 182 brain regions (Fig. 3K, L), including the thalamus, fornix, corpus callosum and regions of the hippocampus (Fig. 3K). The most notable differences were observed in the hypothalamus, medulla, pons, posterior commissure and dentate nucleus (FDR < 0.01, Fig. 3K). Taken together, the dysregulated lipid profiles, increased desmosterol, neuroanatomical changes, impaired neuromuscular function, and heightened startle response in *Osbpl6*^*−/−*^ mice suggest a protective role for ORP6 in the development of AD.

### ORP6 expression is highest in neurons and astrocytes of the CNS

**Fig. 4:**
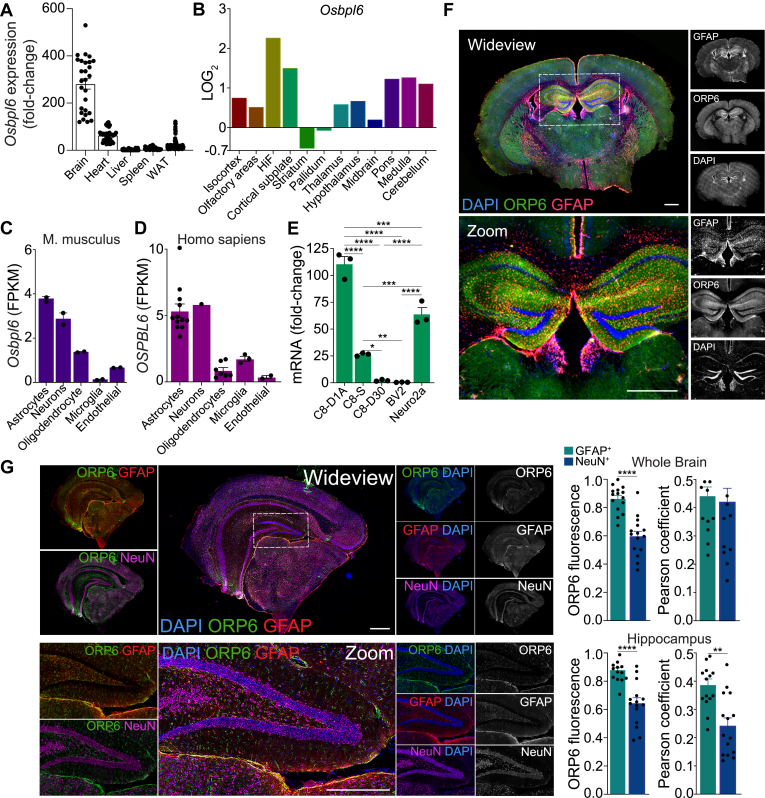
ORP6 is enriched in hippocampal astrocytes. A: *Osbpl6* expression in mouse brain, heart, liver, spleen and white adipose tissue (WAT), expressed as fold change relative to liver, extracted from the mouse gene expression database (GXD) (mean ± s.e.m.). B: *Osbpl6* gene expression (Log_2_ fold-change) in specific brain regions from the Allen Brian Atlas. C, D: Mouse *Osbpl6* and human *OSBPL6* mRNA enrichment in astrocytes and neurons (brainrnaseq.com). E: qRT-PCR of *Osbpl6* mRNA in murine CNS cell lines, normalized to BV2 cells (mean ± s.e.m.). F: Immunofluorescence of GFAP (*red*) and ORP6 (*green*) colocalization (*yellow*) in mouse brain. DAPI is shown in *blue*. G: Immunofluorescence of NeuN (*purple*), GFAP (*red*), DAPI (*blue*), and ORP6 (*green*) with ORP6 fluorescence and Pearson’s correlation for overlap with GFAP^+^ or NeuN^+^ areas (n = 6–8 mice/sex/genotype). Scale bar = 1 mm (F, G). ∗*P* < 0.05, ∗∗*P* < 0.005, ∗∗∗*P* < 0.0005, ∗∗∗∗*P* < 0.00005 by one-way ANOVA with Tukey’s multiple comparisons test (A, C, G).

*Osbpl6* expression analyses revealed that ORP6 was highly enriched in the brain compared to other mouse tissues (Fig. 4A). Additionally, ORP6 exhibited spatial and cell-type-specific expressions within the CNS. Among the various regions of the mouse brain, ORP6 levels were highest in the hippocampal formation (HiF), followed by the cortical subplate, pons, medulla and cerebellum (Fig. 4B). In both mouse and human brains, ORP6 expression was highly enriched in neurons and astrocytes (Fig. 4C, D). To further elucidate the cell-specific expression of ORP6 in the CNS, we quantified *Osbpl6* mRNA across several immortalized mouse cell lines: C8-D1A, C8-D30 and C8-S for types I, II, and III astrocytes respectively; Neuro-2a for neurons; and BV2 for microglia. Quantitative real-time PCR (qRT-PCR) revealed enrichment of *Ospbl6* mRNA in neurons and astrocytes relative to microglia, with the highest expression in type I astrocytes (Fig. 4E). Immunofluorescent staining for ORP6 revealed a widespread expression of ORP6 across various brain regions, including the hippocampus (Fig. 4F). In the whole brain and the hippocampus, ORP6 was abundant in GFAP^+^(astrocytes) and NeuN^+^(neurons) positive areas, with a higher abundance in astrocytes compared to neurons (Fig. 4G). Within the hippocampus, ORP6 colocalized more extensively with the astrocyte marker GFAP than with NeuN (Fig. 4G). Collectively, these data point to a physiological role for ORP6 in hippocampal astrocytes within the CNS.

### ORP6 regulates astrocyte cholesterol homeostasis

**Fig. 5:**
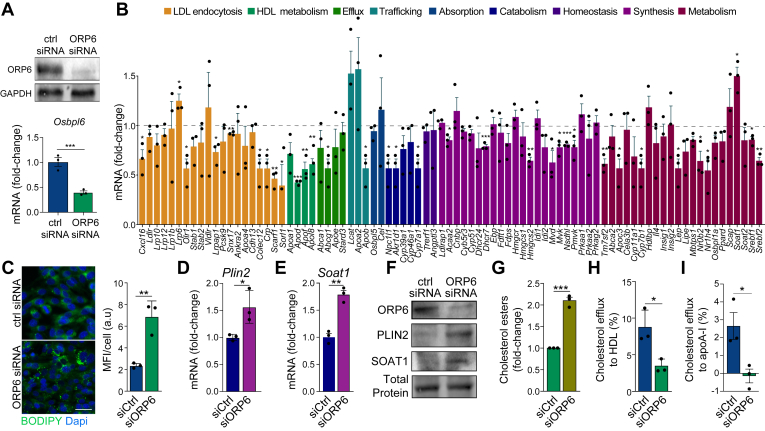
ORP6 regulates cholesterol homeostasis in astrocytes. A: Immunoblot and qRT-PCR for ORP6 in C8-D1A astrocytic cells treated with control (ctrl) or *Osbpl6* siRNA. B: qRT-PCR of lipid metabolism genes in astrocytes treated as in (A), showing fold-change relative to control (mean ± s.e.m.). C: immunofluorescence staining for BODIPY (*green*) and DAPI (*blue*) in C8-D1A cells treated with ctrl siRNA or ORP6 siRNA. D, E: qRT-PCR of *Plin2* and *Soat1* mRNA in ORP6 versus control siRNA-treated C8-D1A cells (n = 5). F: immunoblot of ORP6, PLIN2, SOAT1 in ORP6 versus control siRNA-treated C8-D1A cells. G: Cholesterol ester quantification by thin layer chromatography in cells with control or ORP6 siRNA loaded with ^3^H-cholesterol for 24 h and effluxed to BSA (n = 3). H, I: ^3^H-cholesterol efflux to HDL (H) or apoA-1 (I) in control or ORP6 siRNA-treated cells over 4h. A–I: Data are mean ± s.e.m., ∗*P* < 0.05, ∗∗*P* < 0.005, ∗∗∗*P* < 0.0005 by Student’s *t* test (A, D, E, G–I) or by one-way ANOVA with Tukey’s multiple comparisons test (B). Scale bar = 25 μm.

Given that astrocytes provide the majority of *de novo*-synthesized cholesterol in the brain, we next investigated the role of ORP6 in glial cholesterol homeostasis. To identify lipid metabolism genes and pathways regulated by ORP6 in astrocytes, we performed gene expression profiling of C8-D1A cells treated with either control or *Osbpl6* siRNA. Our results showed that *Osbpl6* siRNA effectively reduced ORP6 mRNA and protein by >80% (Fig. 5A). Knockdown of *Osbpl6* resulted in decreased expression of cholesterol biosynthesis genes, including 7-dehydrocholesterol reductase (*Dhcr7*), 3-Hydroxy-3-Methylglutaryl-CoA Synthase 1 (*Hmgcs1*), and Sterol-4-alpha-carboxylate 3-dehydrogenase (*Nsdhl*) (Fig. 5B). Additionally, genes involved in HDL metabolism, sus as *Apod*, *Apof*, *Apol8*, and the cholesterol transporter *Abcg1*, were downregulated (Fig. 5B). Expression of cholesterol catabolism enzymes cholesterol 7 alpha-hydroxylase (*Cyp7a1*) and oxysterol 7-alpha-hydroxylase (*Cyp7b1*), as well as the lipogenic regulators sterol regulatory element binding proteins 1 and 2 mRNA (*Srebf1*, *Srebf2*), was also reduced (Fig. 5B). In contrast, LDL receptor related protein 6 (*Lrp6*), a regulator of cholesterol homeostasis (63), and sterol O-acyltransferase 1 (*Soat1*), which encodes ER-resident acyl-coenzyme A:cholesterol acyltransferase (ACAT) for cholesterol esterification (64), were upregulated (Fig. 5B). These findings indicate that silencing ORP6 in the C8-D1A astrocyte cell line leads to significant alterations in gene expression related to cholesterol biosynthesis, efflux, and esterification.

### ORP6 knockdown in astrocytes promotes lipid droplet accumulation

Notably, ORP6 knockdown in C8-D1A cells resulted in a marked increase in neutral lipids compared to control siRNA-treated cells, as detected by BODIPY staining (Fig. 5C). Increased accumulation of neutral lipids in C8-D1A cells upon ORP6 knockdown was associated with elevated mRNA expression of the LD marker perilipin 2 (*Plin2*) (Fig. 5D) and the cholesterol esterification enzyme *Soat1* (Fig. 5E) by qRT-PCR. Increased expression of PLIN2 and SOAT1 (better known as ACAT1) was confirmed at the protein level by Western blot analysis (Fig. 5F). To quantify cholesterol esterification, we pulsed control or ORP6 siRNA-treated C8-D1A cells with ^3^H-cholesterol to subsequently quantify its incorporation into ^3^H-cholesterol esters. Knockdown of ORP6 resulted in a two-fold increase in ^3^H-cholesterol esters (Fig. 5G). OPR6 knockdown reduced ^3^H-cholesterol efflux to both HDL and lipid-poor apolipoprotein A-I (apoA-I) (Fig. 5H, I). Taken together, our results show an important role for ORP6 in regulating the esterification of astrocyte cholesterol, LD biogenesis, and cholesterol efflux.

### ORP6 knockdown in mouse astrocytes reduces plasma membrane cholesterol and promotes APP processing and AβO production

**Fig. 6:**
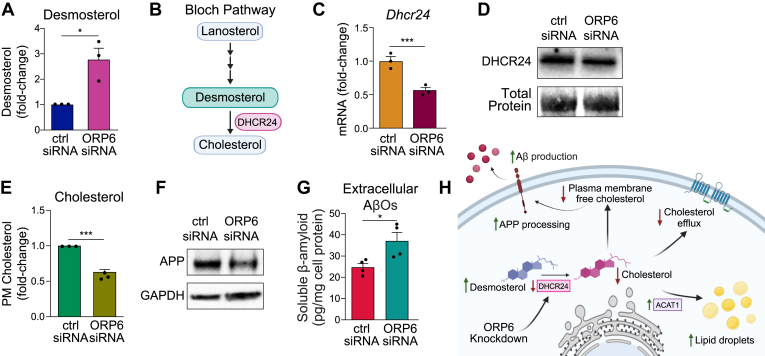
ORP6 knockdown enhances amyloid-beta production by lowering plasma membrane cholesterol and increasing APP processing. A: Relative desmosterol content in C8-D1A cells transfected with ctrl or ORP6 siRNA and loaded with ^3^H-mevalonate for 24 h (n = 3, mean ± s.e.m). B: Schematic overview of the Bloch Pathway for cholesterol biosynthesis in astrocytes, highlighting desmosterol as a precursor to cholesterol, catalyzed by DHCR24. C: qRT-PCR of *Dhcr24* mRNA in ORP6 versus ctrl siRNA-treated C8-D1A cells (n = 3 with, mean ± s.e.m.). D: Immunoblotting of DHCR24 in C8-D1A cells transfected with ctrl or ORP6 siRNA. E: Quantification of de novo synthesized plasma membrane (PM) ^3^H-cholesterol content extracted from cells radiolabelled as in (A). Cholesterol was extracted from the PM by incubation of cells with 10 mM mβ-CD at 4˚C for 15 min (n = 3, mean ± s.e.m.). F: Immunoblotting of ORP6, full-length amyloid precursor protein (APP) and GAPDH in protein lysates of C8-D1A cells transfected with ctrl or ORP6 siRNA. G: Extracellular amyloid-beta oligomer (AβOs) concentrations (pg/mg protein) in the supernatants of cells treated as in (F) (n = 3, mean ± s.e.m.). H: Model: ORP6 knockdown decreases DHCR24 and increases ACAT1, leading to esterified cholesterol accumulation, reduced cholesterol efflux, lower plasma membrane cholesterol, and increased APP processing and neurotoxic AβO production. ∗*P* < 0.05, ∗∗*P* < 0.005, ∗∗∗*P* < 0.0005 by Student’s *t* test (A, C, E, G, H).

In the CNS, amyloid beta peptides arise from amyloid precursor protein (APP) cleavage by α- or β-secretase enzymes that generate αAPP or βAPP and the shorter α-cleaved COOH-terminal stub of APP subsequently cleaved by γ-secretases (65). All these enzymes localize to cholesterol-rich membrane domains (65, 66, 67, 68), making cholesterol concentrations in these microdomains crucial for APP processing. Dysregulated cholesterol trafficking in the CNS contributes to AD development (69). Given that ORP6 regulates intracellular cholesterol trafficking (6) and that increased desmosterol levels were found in the brains of *Osbpl6*^*−/−*^mice (Fig. 2F), we tested whether altered plasma membrane cholesterol in astrocytes lacking ORP6 affects APP processing and AβO production. Intracellular desmosterol levels were markedly increased in C8-D1A astrocytes after ORP6 knockdown (Fig. 6A), suggesting reduced activity of 3β-hydroxysterol Δ(24)-reductase (DHCR24), which converts desmosterol to cholesterol (Fig. 6B). Indeed, DHCR24 expression decreased at both the mRNA and protein levels in cells treated with ORP6 siRNA compared to controls (Fig. 6C, days). In contrast, while ORP6 silencing reduced mRNA expression of *Dhcr7* as compared to controls, DHCR7 protein levels remained unchanged (Supplementary Fig. 1). ORP6 knockdown reduced plasma membrane cholesterol content (Fig. 6E) and increased APP processing, evidenced by decreased full-length APP levels (Fig. 6F), and increased extracellular soluble Aβ oligomers (Fig. 6G). Together, these results indicate that ORP6 regulates plasma membrane cholesterol content, APP processing, and soluble Aβ oligomer production in mouse astrocytes (Fig. 6H).

## Discussion

While cholesterol homeostasis is fundamental to human health, our understanding of cholesterol trafficking between cell compartments remains incomplete. Fluctuations in cellular cholesterol concentrations are sensed by SREBPs and the sterol responsive LXRs that coordinate the expression of cholesterol biosynthesis, uptake and efflux genes. These intricate cholesterol-sensing pathways entail coordinated inter-membrane cholesterol transport, facilitated by lipid transfer proteins (LTPs). To date, several LTPs exhibit the capacity for mediating sterol transfer in vitro yet assigning physiologic roles for these remains challenging. Only a handful of intracellular LTPs were attributed clear physiologic functions in vivo: Niemann Pick Type C proteins 1 and 2 (NPC1, NPC2) that are required for lysosome cholesterol export (69), steroid acute regulatory protein (STARD1) that is required for cholesterol traffic to the inner mitochondrial membrane (70, 71, 72), and more recently Aster proteins that shuttle cholesterol between ER-plasma membrane contact sites (73). Here, we identify LXR-regulated ORP6, one of the least studied members of the ORP family of LTPs, as a critical regulator of lipid metabolism.

Most of our insights into the structural and functional roles of ORPs come from *Saccharomyces cerevisiae* OSBP homologues (Osh) proteins, Osh1p-Osh7p (74). In yeast, the seven Osh proteins are functionally redundant, where none are singularly required for viability (75). However, the collective mutation of all Osh proteins is lethal (75). Similarly, the collective deletion of all *Caenorhabditis elegans* homologs, OBR-1 to OBR-4, is embryonic lethal (76). Understanding ORP functions in mice has proven difficult due to a lack of available genetic mouse models; attempts to delete OSBP in mice proved fatal at early embryonic stages, highlighting its critical role but leaving its physiologic function unknown (77). The first viable ORP knockout mouse model was only recently developed, with systemic ORP8 deletion leading to elevated HDL-cholesterol and sex- and diet-specific lipid metabolism changes (78). In turn, ORP8 deletion in the bone marrow of hypercholesterolemic *Ldlr*^*−/−*^ mice reduced atherosclerosis (79). Our generation of *Osbpl6*^*−/−*^ mice thus introduces the second viable ORP knockout mouse.

In our studies, ORP6 was highly enriched in the brain, with abundant expression within the hippocampus, a region critical for memory formation and spatial navigation. ORP6 was highly localized to astrocytes, underscoring its role in astrocytic lipid metabolism. Remarkably, ORP6 deletion alone was sufficient to drive neurotoxic Aβ oligomer production in mice without aging or dietary interventions. A growing body of evidence suggests that AβO accumulation can singularly drive the development of AD-related learning and memory deficits (80). Correspondingly, *Osbpl6*^*−/−*^ mice showed early signs of AD, such as reduced grip strength (57, 58) and altered axon and glutamate receptor pathway expression (81, 82).

In a family-based genetic study, OSBPL6 variants (rs1347297, rs72953347) were linked to increased AD risk (83), though their impact on ORP6 expression remains unknown. In AD patients, ORP6 expression was strikingly reduced in astrocytes (56). In APP/PS1 mice, which overproduce Aβ and are used in studies of AD pathogenesis (45), ORP6 levels in the hippocampus were also lower at both the mRNA and protein levels. The mechanism behind ORP6 dysregulation in AD remains unknown. However, recent findings reveal that miR-33, a key regulator of lipid metabolism and co-transcriptional product of SREBP-2, can drive AD by promoting Aβ secretion and impairing its clearance (84). Conversely, miR-33 knockdown protects against Aβ-induced inflammation, oxidative stress, apoptosis, and synaptic damage (85). Given that SREBP-2 nuclear activity in the CNS is elevated in AD (86), driving a feed-forward positive regulatory loop with miR-33, and that ORP6 is a major miR-33 target, reduced ORP6 expression in AD might be due to elevated SREBP-2/miR-33 expression. This reduction in ORP6 could disrupt cholesterol esterification, leading to an accumulation of cholesterol esters that can promote the production of AβOs (86). Interestingly, inhibition of ACAT1, which we found markedly increased upon ORP6 knockdown, can ameliorate amyloid pathology in mice with AD (87), suggesting that restoring ORP6 expression may reverse AD-related lipid dysmetabolism.

Lipid dysregulation is a hallmark of AD pathogenesis (88, 89), often evident in altered plasma lipid profiles and brain cholesterol metabolism genes, which can precede detectable cognitive and neuropathological changes (90, 91, 92). In astrocytes, ORP6 knockdown reduced HDL metabolism gene expression, including *Apod*, *Apof*, *Apol8*, and *Abcg1*, while *Osbpl6*^*−/−*^ mice showed reduced plasma HDL-C levels. These findings align with evidence linking the *OSBPL6* gene locus to HDL levels and premature coronary artery disease (6, 93, 94). Other ORP members also influence lipid levels; ORP8 affects HDL-C, and ORP10 knockdown enhances triglyceride synthesis and secretion (95). SNPs in ORP7, OSBPL11, and OSBPL10 have been linked to LDL-C levels, hyperglycemia, and cardiovascular risk factors (96, 97, 98).

Emerging evidence points to circulating triglycerides, cholesterol, HDL, and LDL plasma levels as potential biomarkers for AD (99, 100, 101, 102, 103). Clinical studies reveal that patients with elevated cholesterol have increased susceptibility to AD (104, 105, 106) and that lowering plasma cholesterol with statin therapy during middle age might confers benefits to cognitive function and reduces AD prevalence (107, 108). In addition, experimental studies using rabbits and a transgenic mouse model of AD (109, 110, 111, 112) have demonstrated that hypercholesterolemia induced by high-fat diet feeding increases Aβ levels in the brain and accelerates extracellular Aβ deposition, driving subsequent cognitive deficits and memory impairments. Alterations in plasma lipid profiles and local brain lipid dysmetabolism, evident in *Osbpl6*^*−/−*^ mice, may underlie the observed neurological changes, particularly considering the established connection between dyslipidemia, blood brain barrier integrity, and neurodegenerative processes (112, 113). Alternatively, changes in brain physiology may reciprocally impact lipid metabolism. Further mechanistic studies will be necessary to define the directionality and causal relationships between these processes more clearly.

While neurons are traditionally considered the principal source of brain Aβ, astrocytes—which outnumber neurons by approximately fivefold—may also contribute substantially to Aβ in AD (114, 115). Our study identifies a novel role for ORP6 in astrocytic APP processing and amyloidogenic regulation. Specifically, ORP6 knockdown in astrocytes reduces DHCR24 expression, resulting in desmosterol accumulation and decreased plasma membrane cholesterol levels, conditions known to promote APP processing and AβO production. We focused on DHCR24 and the Bloch pathway because ORP6 knockdown in astrocytes selectively reduced DHCR24 protein levels, while DHCR7 protein remained unchanged, consistent with impaired Bloch pathway flux. Although the Kandutsch-Russell pathway predominates in neurons, astrocytes primarily rely on the Bloch pathway for cholesterol biosynthesis (116, 117, 118, 119). Accordingly, our model in Figure 6 reflects astrocyte-specific cholesterol metabolism, highlighting ORP6 as a regulator of DHCR24. Given previous studies implicating DHCR24 in modulating APP processing and Aβ production (120, 121, 122, 123), we propose that restoring DHCR24 function may counteract the APP accumulation observed in ORP6-deficient astrocytes. Future studies will aim to validate the causal role of DHCR24 downregulation in mediating the ORP6 deficiency phenotype.

Desmosterol, the immediate cholesterol precursor elevated upon ORP6 knockdown, is also a potent endogenous agonist of LXRs (124), which regulate cholesterol homeostasis and inflammation. Although LXR activity was not directly assessed in this study, the accumulation of desmosterol suggests a shift in LXR-regulated signaling. Notably, despite increased desmosterol levels in AD (49), LXR activity may be paradoxically impaired in AD (125), indicating a possible decoupling of ligand availability and receptor function. This dysfunction may contribute to lipid and immune dysregulation associated with ORP6 deficiency. Moreover, diminished LXR activity in AD could also account for the reduced ORP6 expression observed in both humans and mice, potentially establishing a maladaptive feedforward loop.

Since the discovery of LTPs more than 50 years ago, research has unveiled their essential roles in cholesterol metabolism. However, how dysregulated cholesterol transport in the brain contributes to neurodegeneration is not well understood. Notably, STARD1 overexpression correlates with Aβ deposition and cholesterol buildup in AD models (72), while NPC1 levels are elevated in the hippocampus and frontal cortex of both AD patients and mouse models (126). These observations emphasize the importance of understanding the physiological roles of LTPs in cholesterol trafficking. Our findings establish ORP6 as a key regulator of cellular and systemic metabolism, revealing its role in modulating the production of neurotoxic AβOs in astrocytes and highlighting it as a promising target for advancing our understanding of, and potentially improving, cognitive health.

## Data availability

Data that support the findings of this study are available from the corresponding author upon reasonable request. The mass spectrometry proteomics data have been deposited to the ProteomeXchange Consortium via the PRIDE (127) partner repository with the dataset identifier PXD035149.

## Supplemental data

This article contains supplemental data.

## Conflict of interest

The authors declare that they have no conflicts of interest with the contents of this article.
