# Supplementary material for: Oxysterol-binding protein ORP6 regulates lipid metabolism and brain Aβ production
Source: J Lipid Res. 2025 Jul 25;66(9):100868. doi: 10.1016/j.jlr.2025.100868 (PMC12455110; doi:10.1016/j.jlr.2025.100868)
Supplement: Supplementary Material [file mmc1.docx]

**Supplementary Figure 1.** **ORP6 knockdown decreases *Dhcr7* mRNA levels without altering DHCR7 protein expression.**

(**A**) qRT-PCR of *Dhcr7* mRNA in ORP6 vs. ctrl siRNA-treated C8-D1A cells (n=4 with, mean ± s.e.m.). (**B**) Immunoblotting of DHCR7 and GAPDH in C8-D1A cells transfected with ctrl or ORP6 siRNA. **P<0.005 by Student’s t-test (A).

**Supplementary Table 1.** Plasma MS signal entities identified by using MS/MS analysis and the Montreal Heart Institute Metabolomic platform internal database.

**Supplementary Table 2.** Brain MS signal entities identified using MS/MS analysis.

**Supplementary Table 3.** Altered biological processes, cell components and molecular functions in WT and *Osbpl6^-/-^* brains.

**Supplementary Table 4.** Sequences used in *Osbpl6^-/-^* mouse generation/genotyping*.

| Name | Sequence (5’-3’) | Target site |
| --- | --- | --- |
| Osbpl6_gRNA_E10U | TGATCATGGTTCCGCCCTCA | Chr2: 76385103-76385122 (-1) |
| Osbpl6_gRNA_E10D | ATTCTTCTAGGCGTAACAAT | Chr2: 76385709-76385728 (+1) |
| ploxP | GCTAGGCTCCTAGGAATAAAACCACAAGCCTACCAAACTCCCCAGAGTGCCCATGAGATGAAATCCGTGAataacttcgtatagcatacattatacgaagttatggtctgagctcgccatcGGGCGGAACCATGATCACACACAATGGGAAGTGTTAATGGTATCTACTCTCTGAGTCATAGTTGGTAAGA | Chr2: 76385036-76385175 (+1) |
| dloxP | tacagaggacacaggtttgattcccagcacccacaaagcagctcacaacaacaataagtggttacctattataacttcgtataatgtatgctatacgaagttatcattaattgcgttgcgcgttacgcctagaagaatccaatgccttcttcgggactgcacagctactgcacacacatggtgcacacata | Chr2: 76385656-76385795 (-1) |
| Osbpl6_wt_F1 | AAAGGGAAGTTACTATTCACAGGCT | Chr2: 76385550-76385574 (+1) |
| Osbpl6_wt_R1 | AATACCAAAGAGAACTGGGAGTTGA | Chr2: 76385913-76385937 (-1) |
| Osbpl6_wtF_F1 | TGCATGAAACCAGGCCTTAGATATA | Chr2: 76384790-76384814 (+1) |
| Osbpl6_wtF_R1 | GAGTTCTCTGAAGGATCTCCAAGTT | Chr2: 76385408-76385432 (-1) |
| ***Genotyping reactions:**  *Osbpl6* wild-type (flanks ploxP) = wtF_F1 + wtF_R1 = 643 bp wild-type and 694 bp em2  *Osbpl6* wild-type (flanks dloxP) = wt_F1 + wt_R1 = 388 bp wild-type and 439 bp em2  *Osbpl6* em1 (*Osbpl6^-/-^*) = wtF_F1 + wt_R1 = 698 bp em1 & 1,148 bp wild-type | | |
